# How to implement medical evidence into practice in developing countries

**DOI:** 10.5116/ijme.57b8.9002

**Published:** 2016-10-02

**Authors:** Manar Al-lawama

**Affiliations:** 1 Department of Pediatrics, University of Jordan, Amman, Jordan

**Keywords:** Medical evidence, practice, developing countries, Jordan

## Introduction

Sackett and colleagues first defined evidence-based medicine as “the conscientious, explicit, and judicious use of current best evidence in making decisions about the care of individual patients”.^1^ Integration of the best research evidence with clinical expertise and patient values is the key element for evidence-based practice. Good judgment is crucial when it comes to evaluating the evidence and implementing it.^2^ Evidence-based medicine is not “one size fits all”, as the world is highly diverse concerning patterns of diseases, economy, and societal values. This article documents the peculiarities of developing countries regarding evidence-based practice and presents a simple education model for the transformation of evidence into practice for undergraduate and postgraduate students.

### Developing countries and evidence-based medicine

Developing countries have multiple issues when it comes to evidence-based medicine. First of all, most of the evidence is imported. If we take all Arab countries as an example, their medical research production over an 18-year period is just 3% of the USA’s production.^3^ Therefore, the best scenario is that 97% of their practice is based on evidence that was not specifically generated for them. This is unsatisfactory as every society, ethnic group, or even culture has its unique diseases or different forms of the same disease, different responses to therapeutic modalities, different resources and personnel qualifications, and perhaps also different values and patient preferences. Secondly, critical appraisal skills are poorly developed among health care professionals^4^ and their incorporation into the curriculums of medical schools is limited.^5^ Consequently, this may result in the adoption of unnecessary practice or improper use of the evidence. Furthermore, it may lead to “jerky medicine syndrome”, the term I use when physicians frequently change their way of practice in response to their latest reading of the literature. Ideally, the medical practice should be consistent with few sudden changes, otherwise achieving competence among the staff will be very difficult and could potentially lead to increased patient morbidity. The final issue regarding practicing medicine in developing countries is that the regulatory bodies of medical practice are less well established and not as empowered. As a consequence, national guidelines are either absent, unclear, or copied from international guidelines.

### Improving evidence-based practice through medical education

To improve the health care in our region of the world, we must invest majorly in our future doctors. Currently, our medical education focuses mainly on medical knowledge. However, because of the vast amount of medical literature published daily, we need to place much greater emphasis on evidence-based medicine. The transformation of research into practice should be a defined structured multistage process that every student or trainee is familiar with, otherwise confusion will reign due to imported medical literature and the eagerness of local physicians to copy the Western medical model.

Our students should learn how to read the evidence. Critical appraisal is one of the most important skills we should teach our students and residents.  A crucial part of the critical appraisal process is whether the evidence is applicable in a particular setting. In addition to similarities in population and rates of illness, several other factors should be considered, e.g. nurse-to-patient ratio, the availability of other medical personnel and their skills level, the availability of devices, and very importantly the compatibility of the evidence with the society’s cultural beliefs and patient preferences.

This article proposes a simple educational model for the transformation of the medical evidence from a published article into medical practice. It can be easily incorporated into the curriculums of courses designed for such purposes. It consists of three stages. The first stage (Publish stage) focuses on identifying the published research with the caliber to drive practice re-evaluation. Physicians should not revise their practice every time a new study is published. They should only do so in response to the publication of a large well-conducted study with major results or multiple studies with significant results reporting harm of a current practice or benefit of a new practice. General critical appraisal skills judging the quality of the evidence should be applied during this stage. The second stage (READI stage: Read, Adopt, and Distribute) is primarily the preparation of the institution for the implementation of the new practice. This stage consists of reading the literature critically, evaluating its applicability locally, economically, and culturally, and subsequently adopting it if applicable. A working plan should be prepared that includes when the practice should start, the requirements needed for the practice on manpower, skills, and devices, and the first evaluation date. Distribution of the necessary knowledge in the form of lectures, posters, and workshops should then follow. Finally, any staff concerns should be addressed prior to introducing the practice. The third stage (Practice stage) is the initiation of the new practice with continuous evaluation. This includes reporting of unexpected mortality, major side effects, and staff confusion. It should also include a structured evaluation after completion of the predetermined period.

## Conclusions

Application of the proposed model has the potential to change our medical culture, especially in our region of the world where physicians are perceived as “up-to-date” based on their eagerness for any emerging evidence and their haste to change their practices. It is hoped that some of the wisdom and soul of medicine will be regained through the teaching of the Publish-READI-Practice model.

### Acknowledgements

I would like to thank Dr Eyad Altamimi for revising this manuscript.

### Conflicts of Interest

The author declares that she has no conflict of interest.
